# Comparative study between apocynin and protocatechuic acid regarding antioxidant capacity and vascular effects

**DOI:** 10.3389/fphys.2022.1047916

**Published:** 2022-11-15

**Authors:** Murilo E. Graton, Bruno H. S. H. Ferreira, Jéssica A. Troiano, Simone R. Potje, Gabriel T. Vale, Ana Cláudia M. S. Nakamune, Carlos R. Tirapelli, Francis J. Miller, Valdecir F. Ximenes, Cristina Antoniali

**Affiliations:** ^1^ Programa de Pós-graduação Multicêntrico em Ciências Fisiológicas, SBFis, São Paulo State University (UNESP), Araçatuba, São Paulo, Brazil; ^2^ Department of Basic Sciences, School of Dentistry, São Paulo State University (UNESP), Araçatuba, São Paulo, Brazil; ^3^ Department of Support, Production and Animal Health, School of Veterinary Medicine, São Paulo State University (UNESP), Araçatuba, São Paulo, Brazil; ^4^ Fundação Dracenense de Educação e Cultura (FUNDEC), Faculdades de Dracena (UNIFADRA), Dracena, São Paulo, Brazil; ^5^ Department of Biosciences, Minas Gerais State University (UEMG), Belo Horizonte, Minas Gerais, Brazil; ^6^ Department of Psychiatry Nursing and Human Sciences, College of Nursing of Ribeirão Preto, University of São Paulo (USP), Ribeirão Preto, São Paulo, Brazil; ^7^ Nashville VA Medical Center, Vanderbilt University, Nashville, TN, United States; ^8^ Department of Chemistry, Faculty of Sciences, São Paulo State University (UNESP), Bauru, São Paulo, Brazil

**Keywords:** apocynin, protocatechuic acid, reactive oxygen species, oxidative stress, nitric oxide

## Abstract

Reactive oxygen species (ROS) derived from NOX enzymes activity play an important role in the development of cardiovascular diseases. Compounds able to decrease oxidative stress damage are potential candidates as drugs and/or supplements for hypertension treatment. Here, we aimed to compare *in vitro* ROS scavenging potency, effective NOX inhibition and effects on vascular reactivity of apocynin to another phenolic compound, protocatechuic acid, in vascular cells from spontaneously hypertensive rat (SHR), where redox signaling is altered and contributes to the development and/or maintenance of hypertension. We evaluated the *in vitro* antioxidant capacity and free radical scavenging capacity of both phenolic compounds. Moreover, we investigated the effect of both compounds on lipid peroxidation, lucigenin chemiluminescence, nitric oxide (NO^•^) levels and ROS concentration in vascular cells of SHR or human umbilical vein endothelial cell (HUVEC). Apocynin and protocatechuic acid presented antioxidant capacity and ability as free radical scavengers, decreased thiobarbituric acid reactive substances (TBARS) in aortic cells from SHR, and increased NO^•^ concentration in isolated HUVEC. Both compounds were able to reduce lucigenin chemiluminescence and increased the potency of acetylcholine in aorta of SHR. However, in SHR aortas, only apocynin diminished the contraction induced by phenylephrine. In conclusion, these results strongly reinforce the potential application of substances such as apocynin and protocatechuic acid that combine abilities as scavenging and/or prevention of ROS generation, establishment of NO bioactivity and modulation of vascular reactivity. Due to its phytochemical origin and low toxicity, its potential therapeutic use in vascular diseases should be considered.

## 1 Introduction

ROS are metabolites from molecular oxygen as a result of normal cellular metabolism, and are essential signaling molecules that regulate various physiological processes, such as activation of transcription factors, kinase phosphorylation, host defense genes, and mobilization of ion transport systems (Touyz and Schiffrin, 2004; Schramm et al., 2012). However, the overproduction of ROS causes adverse modifications in macromolecules, and the development of several diseases (Checa and Aran, 2020). The imbalance between oxidants and antioxidants in favor of oxidants is called oxidative stress (Birben et al., 2012). Oxidative stress contributes for many pathological conditions, such as hypertension (Rodrigo et al., 2011). Hypertension is an important risk factor for the development of cardiovascular diseases (Fuchs and Whelton, 2020), which are the leading cause of deaths worldwide (GBD 2013 Mortality and Causes of Death Collaborators, 2015).

In the vascular system, ROS regulates endothelial function and vascular tone under physiological conditions, but in pathological situations, ROS play an important role in angiogenesis and rarefaction, inflammation, hypertrophy, proliferation, migration, fibrosis, vascular remodeling, and endothelial dysfunction associated with hypertension (Touyz and Briones, 2011). During hypertension, in addition to the multifactorial processes that lead to blood pressure elevation (interaction among multiple organ systems, many genes, environmental stimuli, activation of the renin-angiotensin system, inflammation, and others) (Touyz and Briones, 2011), oxidative stress contributes to excessive ROS production, decreased NO^•^ bioavailability and decreased antioxidant capacity (Checa and Aran, 2020).

There are two ways for the elimination of ROS. The first is their scavenging by the stimulation of endogenous antioxidant systems or through the administration of antioxidants. The second is more complex and involves the inhibition of enzymes that catalyze the production of ROS (Schramm et al., 2012). Nicotinamide adenine dinucleotide phosphate (NAD(P)H) oxidases (NOX) are the major source of ROS in the vascular tissues (Sedeek et al., 2009; Dikalov et al., 2014). NOX family comprises seven isoforms, each based on a distinct core catalytic subunit, and all of them use NADPH as an electron donor and catalyze electrons transfer to molecular oxygen to generate superoxide anion (O_2_^•-^) (Sirker et al., 2011).

Several approaches to overcome oxidative stress in the vascular wall have been studied as a possible treatment for hypertension (Schramm et al., 2012), and the compounds apocynin and protocatechuic acid are potential candidates.

Apocynin is an acetophenone initially extracted from the root extracts of the medicinal herb *Picrorhiza kurroa* in the Himalayan Mountains (Petrônio et al., 2013). Our group has studied the effects of apocynin in normotensive and hypertensive rats. In aortas from Wistar rats, apocynin promoted a long-lasting hypotensive effect, and it was more potent inducing endothelium-dependent and independent relaxation than its active dimer, diapocynin (Potje et al., 2017). Furthermore, in SHR, chronic treatment with apocynin reduced blood pressure and oxidative stress biomarkers, reversed endothelial dysfunction (Perassa et al., 2016), increased NO^•^ production and reduced ROS generation in blood vessels (Graton et al., 2019). Apocynin has a controversial mechanism of action. Some studies suggest that its effects depend only on its antioxidant properties (Heumüller et al., 2008), whereas other studies suggest its ability to inhibit NOX in phagocytic (Simons et al., 1990) and cardiovascular cells (El-Sawalhi and Ahmed, 2014). Protocatechuic acid is a naturally occurring phenolic acid, widely distributed and present in most edible plants used in folk medicine, being one of the biologically active components of some medicinal plants, fruits and honey (Kakkar and Bais, 2014). There is evidence that it reduces the risk of cardiovascular diseases due to its anti-inflammatory, antioxidant and free radical scavenger properties (Semaming et al., 2015).

The aim of this study was to compare the antioxidant and/or NOX inhibitory properties of apocynin with those of protocatechuic acid, and their potential effect in vascular dysfunction in SHR.

## 2 Materials and methods

The Animal Research Ethics Committee of the School of Dentistry of Araçatuba, UNESP, approved all the procedures used in this study (Process CEUA FOA 2016-00130).

### 2.1 Animals

This study used three-month-old male SHR rats with systolic blood pressure (SBP) > 150 mmHg. Age-matched Wistar rats (SBP ≤ 110-112 mmHg) were used as normotensive control. SBP was assessed by tail plethysmography (PowerLab, ADInstruments, Australia). The animals were housed in a thermoneutral environment (22 ± 2°C) on a 12–12 h photoperiod, with free access to standard food and water. Animals were used in the experiments described below.

### 2.2 Evaluation of antioxidant capacity of the compounds

The antioxidant ability of the compounds in solution were measured by ferric reducing antioxidant power assay (FRAP), a method based on reducing the ferric complex tripyridyltriazine to Fe^2+^ in acidic medium (Benzie and Strain, 1996). Apocynin and protocatechuic acid were first diluted in dimethyl sulfoxide, and then diluted in deionized water from 0.1 to 1 mmo/L. Twenty microliters (20 µl) of each sample, in duplicate, were included in the working solution (composition, in mmol/L: acetate buffer 300.0; 2,4,6-tripyridyl-s-triazine 10.0; FeCl_3_ 20.0; 10:1:1, pH 3.6, respectively). After 30 min incubation at 37°C in the dark, absorbance was estimated at 595 nm (Biotek, United States). Results were expressed in mmol FeSO_4_/L; a FeSO_4_ standard curve was used.

### 2.3 Oxygen radical absorbance capacity assay

ORAC values of the studied compounds were estimated as previously described with mild modifications (Ou et al., 2001). Briefly, the reaction mixtures (final volume 200 μl) were composed of 130 μl fluorescein (110 nmol/L), 50 μl 2,2-azobis (2-amidinopropane) dihydrochloride (AAPH, 80 mmol/L) and 20 μl tested compounds or Trolox (reference antioxidant) or phosphate buffered saline (PBS) (control). All the solutions were prepared in PBS. The reaction mixtures were incubated at 37°C, and the fluorescence intensity (470/520 nm) read at 5 min interval for 2.5 h using a plate reader (Synergy H1 Hybrid Reader, Biotek, United States). The values of (F/F0) were plotted as a function of time, and the integration of the area under the curve (AUC) measured and employed to obtain ORAC values, according to the following equation: ORAC = ([AUC tested compound – AUC control]/[AUC Trolox – AUC control]) × ([Trolox]/[tested compound]). All experiments were carried out in triplicate. ORAC values were expressed as Trolox equivalents, which is the concentration of Trolox that causes the same effect of the tested compounds.

### 2.4 Conjugated autoxidizible triene assay

CAT values of the studied compounds were estimated as previously described with mild modifications (Laquerre et al., 2008). Briefly, the reaction mixtures (final volume 250 μl) were composed of 50 μl Tung oil suspension, 50 μl AAPH (5 mmol/L) and 50 μl of tested compounds or Trolox (reference antioxidant) or PBS (control). All the solutions were prepared in PBS. The reaction mixtures were incubated at 37°C and the absorbance (273 nm) read at 5 min interval for 3 h using a plate reader (Synergy H1 Hybrid Reader, Biotek, United States). The values of (A/A0) were plotted as a function of time and the integration of the area under the curve (AUC) measured and employed to obtain CAT values, according to the following equation: CAT = ([AUC tested compound – AUC control])/[AUC Trolox – AUC control]) x ([Trolox]/[tested compound]). All experiments were carried out in triplicate. CAT values were expressed as Trolox equivalent (TE). The emulsion was prepared by mixing 5 mg of Tung oil (not stripped of tocopherols) in 50 ml of PBS containing 17 μmol/L Brij 35. The solution was vigorously vortexed to produce a homogeneous emulsion.

### 2.5 DPPH radical scavenging assay

The reaction mixtures were composed of 50 µl of 2,2′-diphenyl-1-picrylhydrazyl radical (DPPH, 800 μmol/L) in the absence (control) or presence of 50 μl studied compounds, and the volume completed to 200 μl with ethanol. The reaction mixtures were incubated in the dark and the absorbance (517 nm) read after 30 min using a plate reader (Synergy H1 Hybrid Reader, Biotek, United States). All the solutions were prepared in ethanol. The scavenging capacity was calculated using the following equation: % scavenging = ([absorbance of control – absorbance of studied compound]/[absorbance of control]) x 100. Results were expressed as 50% effective concentration (EC_50_), according to Saroni-Arwa et al. (2015). All experiments were carried out in triplicate.

### 2.6 Treatment of the aorta rings

Animals were anesthetized with isoflurane in a saturated chamber and then decapitated. Thoracic aorta was removed, placed in a Petri dish containing cold Krebs solution (mmol/L: NaCl 130.0; KCl 4.7; KH_2_PO_4_ 1.2; MgSO_4_ 1.2; NaHCO_3_ 14.9; C_6_H_12_O_6_ 5.5; CaCl_2_ 1.6; pH 7.4), dissected, and cut into 4-mm rings. Rings were placed for 30 min in Krebs solution, 37°C, bubbled (95% O_2_ and 5% CO_2_) in the absence (Control) or in the presence of apocynin (100 μmol/L) or protocatechuic acid (100 μmol/L). Rings were frozen in liquid nitrogen and stored at −80°C until the experiments. This procedure was performed for TBARS and lucigenin chemiluminescence experiments.

### 2.7 Determination of lipid peroxidation

In the aortic rings (n = 5–7), lipid peroxidation was evaluated by the concentration of TBARS, using a commercial kit (Cayman). According to the manufacturer’s instructions, this assay measures the concentration of malondialdehyde (MDA), a major lipid oxidation product, and some minor related compounds, and it has been considered a good index of the level of oxidative stress in biological samples. Here, thiobarbituric acid reacts with malondialdehyde forming a pink chromogen (TBARS), which is measured at 532–535 nm. Tissues were macerated individually in RIPA buffer plus protease inhibitor and then centrifuged at 1,600×g, for 10 min at 4°C. Results were expressed as nmol MDA/mg protein.

### 2.8 Detection of superoxide anion production

The lucigenin-derived chemiluminescence assay was used to determine superoxide anion production in the rings (n = 9–10) incubated with the studied compounds. Lucigenin, an acridinium dinitrate compound, is reduced by superoxide anion eliciting light emission. It has been used to measure the production of superoxide anions by biological systems, and it is a valid probe for superoxide production by enzymatic and cellular sources (Li et al., 1998). Luminescence was measured in luminometer (Orion II, Berthold Detection Systems, Germany), and results were expressed as relative light units (RLU)/mg protein.

### 2.9 Measurement of [ROS] and [NO] in HUVEC

HUVEC were purchased from ATCC cell lines (United States). Cells were cultured in Dulbecco’s Modified Eagle’s Medium (DMEM) supplemented with fetal bovine serum (FBS) 10% and used at passages 4–6. After serum deprivation in culture medium for 12  h, confluent cells (10^4^ cells/well) (n = 4) were incubated with apocynin or protocatechuic acid at 100 μmol/L or not (control) for 12 h. Then, cells were incubated with the fluorescence probes dihydroethidium (DHE, a ROS selective probe, 2.5 μmol/L), or diaminofluorescein-2 diacetate (DAF-2DA, a NO^•^ selective probe, 5 μmol/L), for 30 min. Fluorometric analyses were performed on a spectrofluorometer (BioTeK^®^) equipped with a 150-W Xenon lamp (Excitation/Emission, in nm: DHE 405/570 and DAF-2DA 488/530).

### 2.10 Vascular reactivity

Thoracic aorta rings (4-mm) from Wistar rats and SHR were placed between two stainless steel stirrups connected to an isometric force transducer (Letica Scientific Instruments, Spain), kept for 60 min of a stabilization period, in Krebs solution, 37°C, pH 7.4, 95% O_2_, and 5% CO_2_. Rings were stretched to a resting tension of 2.0 g, the functional integrity of the rings was tested with KCl 120 mmol/L, and rings that contracted ≥1.5 g were considered viable and used for the study. All aortic rings used in this study had endothelium preserved. Endothelium integrity was tested and confirmed by relaxation with acetylcholine (ACh, 10 μmol/L) after contraction induced with phenylephrine (PE, 1 μmol/L). Only rings with ≥70% relaxation were used in the following protocols.

#### 2.10.1 Evaluation of the effects of phenolic compounds on phenylephrine-induced contraction

Intact aortic rings (n = 4–8) were incubated for 30 min with one of the compounds studied (apocynin or protocatechuic acid, 100 μmol/L) for 30 min. After that, cumulative concentration-response curves to PE (0.1 nmol–0.1 μmol/L) were performed in the presence of the compounds. Rings without incubation of any compounds were used as control.

#### 2.10.2 Evaluation of the effects of phenolic compounds on acetylcholine-induced relaxation

Intact aortic rings (n = 3–7) were incubated for 30 min with apocynin or protocatechuic acid (100 μmol/L), and then contracted with PE (0.1 μmol/L). After the contraction plateau, cumulative concentration-response curves to ACh (0.1 nmol–0.1 μmol/L) were performed. Rings without incubation of any compounds were used as control.

### 2.11 Drugs and reagents

**FIGURE 1:**
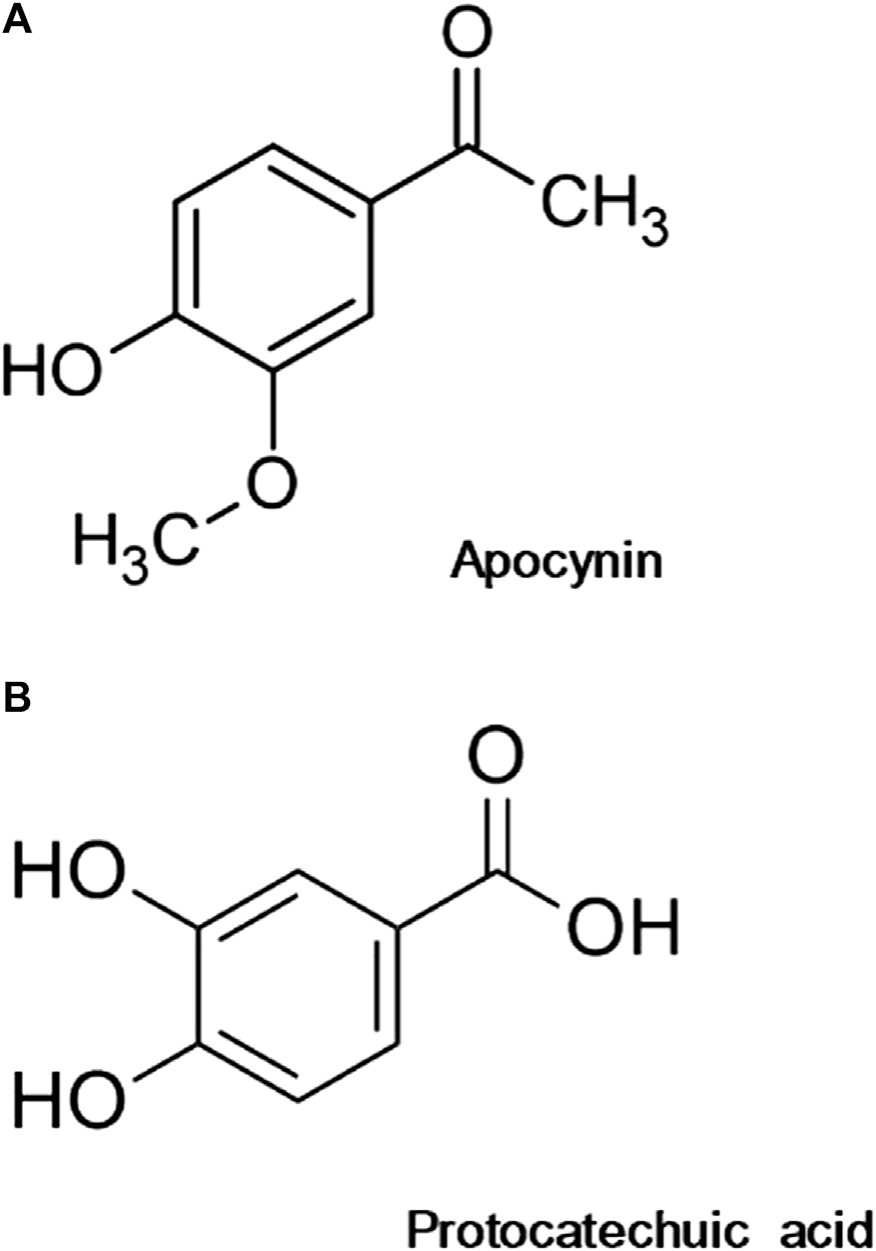
Chemical structure of apocynin **(A)** and protocatechuic acid **(B)**.

Apocynin (C_9_H_1_0O_3_, A10809, Figure 1A), protocatechuic acid (C_7_H_6_O_4_, 37580, Figure 1B), acetylcholine (A6625), phenylephrine (P6126), dihydroethidium (DHE), 4,5-diaminofluorescein diacetate (DAF-2DA), 2,2-Diphenyl-1-picrylhydrazyl (DPPH, D9132), 2,2′-Azobis (2-methylpropionamidine) dihydrochloride (AAPH, 440914) (±)-6-Hydroxy-2,5,7,8-tetramethylchromane-2-carboxylic acid (Trolox, 238,813), Brij 35 (801,962), Tung oil (440,337), and fluorescein (F6377) were acquired from Sigma Aldrich (United States). Lucigenin (L6868) was obtained from ThermoFisher Scientific (United States). DMEM was purchased from Vitrocell (00,025, Brazil) and FBS from Gibco (12657029, South America). TBARS assay kit was acquired from Cayman Chemical (10009055, United States). The remaining salts and reagents were acquired from Sigma Aldrich.

### 2.12 Statistical analysis

Results were expressed as the mean ± standard error of the mean (SEM) or standard deviation (SD), and *n* represents the number of aortas or aorta rings used in the experiments, or the number of independent experiments performed with isolated cells. In vascular function studies, the concentration that produced half-maximal contraction (Phenylephrine) or half-maximal relaxation (Acetylcholine) amplitude (EC_50_) was determined after logarithmic transformation of the normalized concentration-effect curves, and EC_50_ values are reported as the negative logarithm (pD_2_) of the mean of individual values for each aortic ring stimulated. All results were compared between groups by using Student’s t test or analysis of variance (ANOVA) and Tukey post-hoc test. GraphPad Prism 5 or 6 (GraphPad Software Corporation, La Jolla, United States) was applied to analyze results and to make the figures. Differences among groups were considered significant when *p* < 0.05.

## 3 Results

### 3.1 Antioxidant capacity of the studied compounds

**FIGURE 2:**
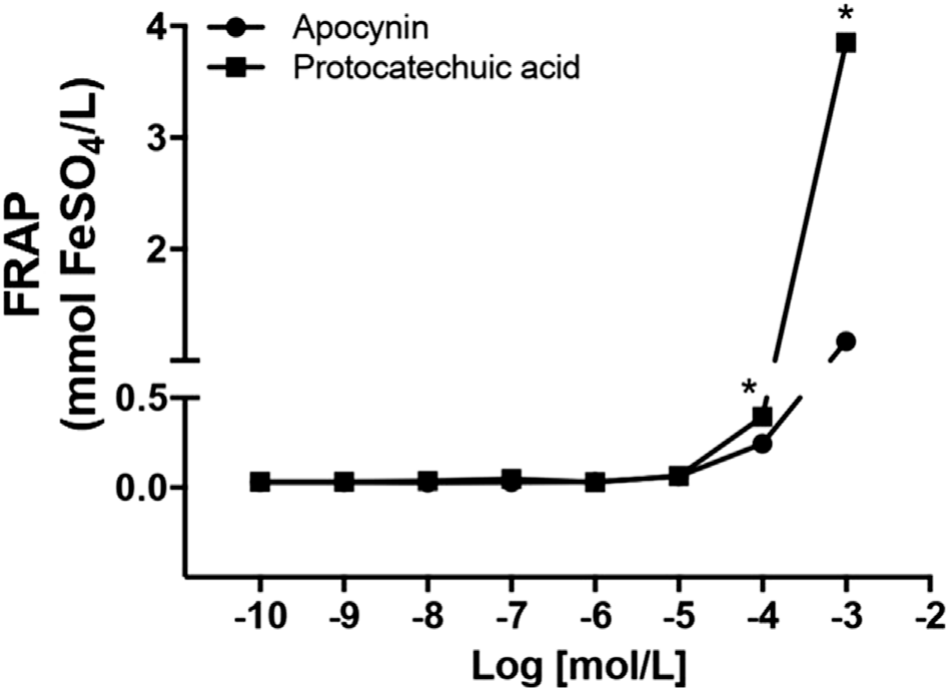
Antioxidant capacity, in mmol FeSO_4_/L, of apocynin (circles) and protocatechuic acid (squares), at concentrations of 0.1 nmol/L to 1 mmol/L *p < 0.05 Protocatechuic acid vs. Apocynin.

Lower concentrations of the apocynin and protocatechuic acid did not demonstrate significant antioxidant capacity (0.1 nmol/L to 10 μmol/L). Antioxidant capacity was observed at concentrations more than 100 μmol/L. In addition, at the concentration of 1 mmol/L protocatechuic acid had higher antioxidant capacity than apocynin (Figure 2; Supplementary Materials).

**FIGURE 3:**
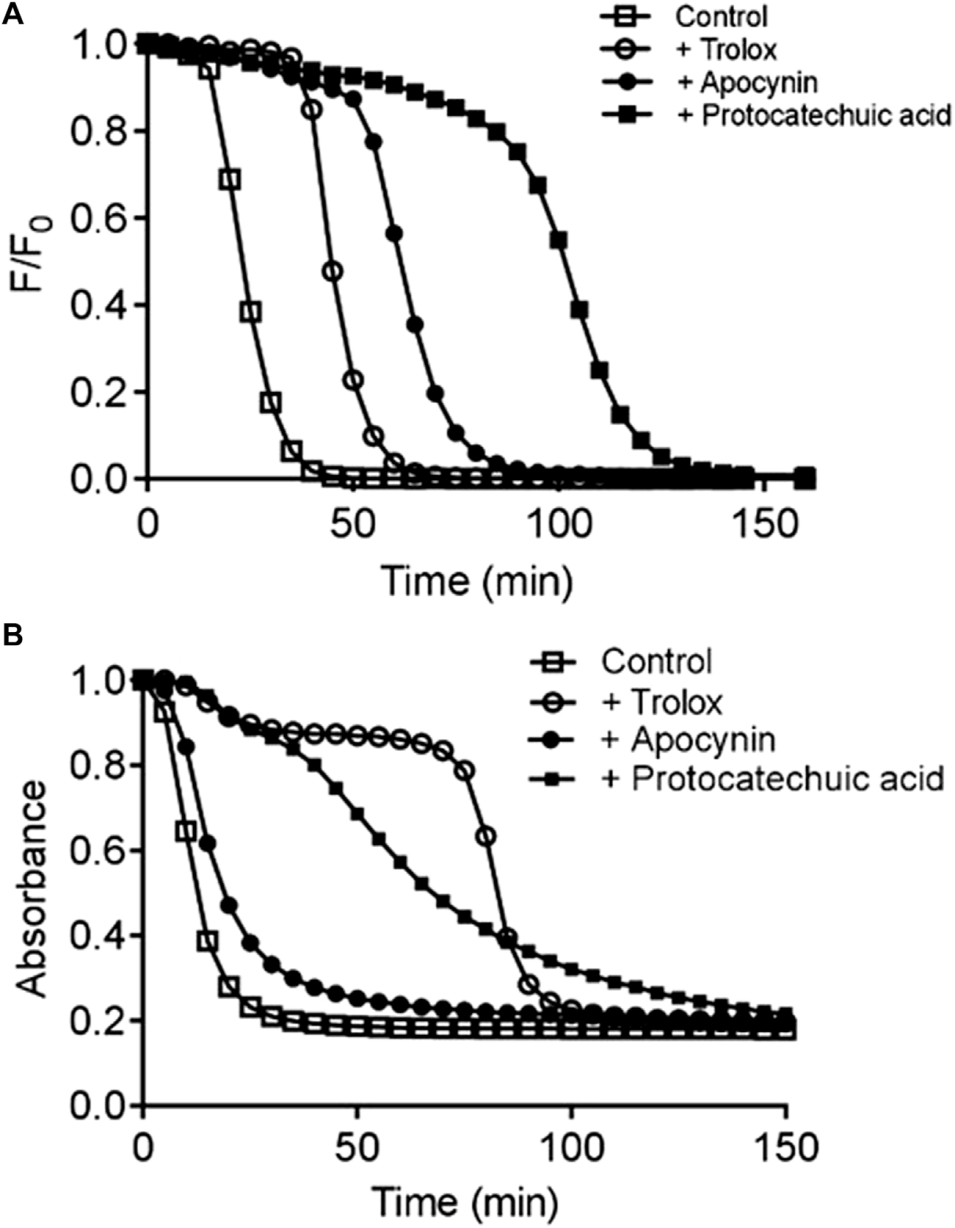
Evaluation of direct free radical scavenging capacity. Kinetic profile of fluorescein degradation by ROO^•^ and the protective effect studied compounds determined by oxygen radical absorbance capacity (ORAC) assay **(A)**. Kinetic profile of triene degradation provoked by AAPH and the protective effect of the studied compounds determined by conjugated autoxidizible triene (CAT) assay **(B)**. ^a^ Trolox equivalent (µmols of Trolox/µmol of studied compound). ^b^ EC_50_ (µmol/L). The results are mean and SD of three experiments. #*p* < 0.05 Protocatechuic acid vs. Apocynin.

Using the oxygen radical absorbance capacity (ORAC) assay, protocatechuic acid showed at least 1.5-fold greater ability to act as a scavenger than apocynin (Table 1 and Figure 3A). Through the CAT degradation assay, protocatechuic acid showed an inhibitory effect six times greater than that of apocynin (Table 1 and Figure 3B). Furthermore, by the DPPH method, protocatechuic acid was significantly more effective than apocynin as it needed at least a 700-fold lower concentration to scavenger DPPH compared to apocynin (Table 1). See description of the results at Supplementary Materials.

**TABLE 1:** Free radical scavenging activity of the studied compounds.

	ORAC^a^	CAT^a^	DPPH (EC_50_)^b^
Apocynin	1.9 ± 0.2^#^	0.11 ± 0.02^#^	19343 ± 2152^#^
Protocatechuic Acid	3.2 ± 0.2	0.63 ± 0.12^#^	26 ± 5

^a^ Trolox equivalent (μmols of Trolox/μmol of studied compound).

^b^ EC_50_ (μmol/L). The results are mean and SD of three experiments. #p< 0.05 Protocatechuic acid *versus* Apocynin.

### 3.2 Apocynin and protocatechuic acid can decrease oxidative stress biomarkers in SHR

**FIGURE 4:**
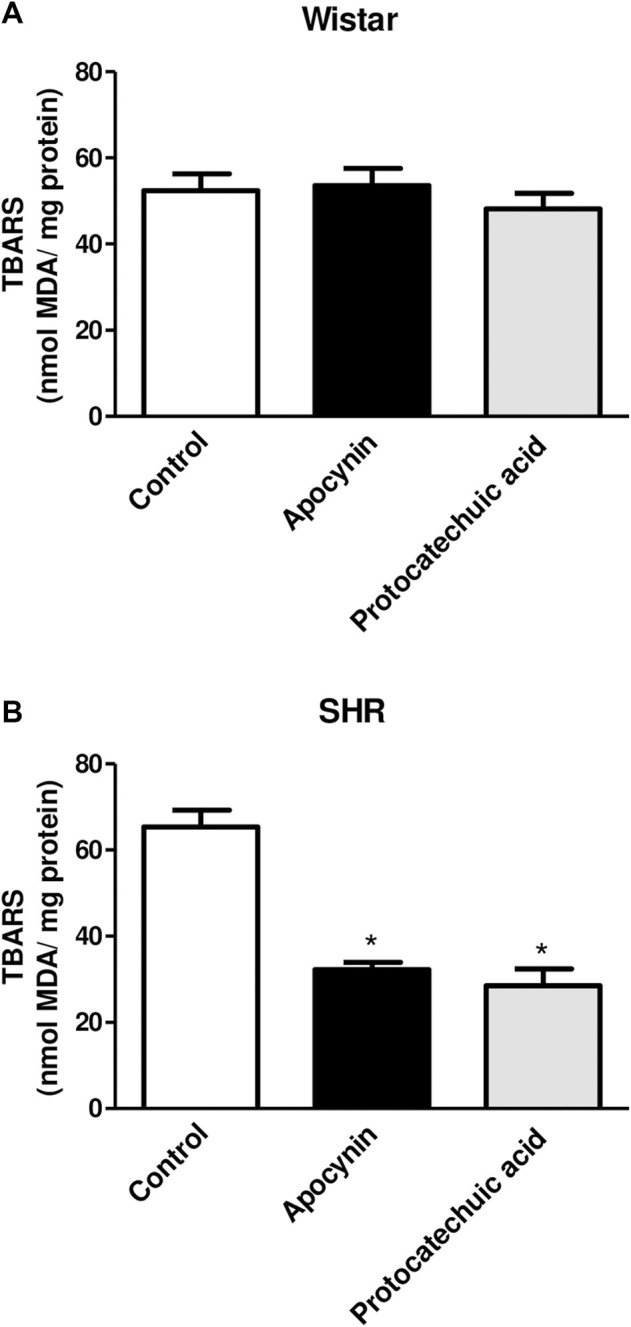
Determination of the percentage of thiobarbituric acid reactive substances (TBARS), in nmol MDA/mg protein, in aorta rings unstimulated (Control, white bars), or stimulated (100 μmol/L) with apocynin (black bars) or protocatechuic acid (grey bars) from Wistar rats **(A)** and SHR **(B)**. Values represent the mean ± SEM of the results, n = 5–7. *p < 0.05 Apocynin and Protocatechuic acid vs. Control.

MDA levels were increased in aortic rings of SHR compared to aortic rings of Wistar rats, indicating oxidative stress in this model (Figure 4; Supplementary Materials). Apocynin and protocatechuic acid did not change MDA levels in aortic rings from Wistar rats, values remaining similar to unstimulated aortic rings (Figure 4A). On the other hand, apocynin and protocatechuic acid were able to reduce MDA levels only in aortic rings from SHR when compared to unstimulated rings (Figure 4B).

### 3.3 Both compounds can decrease ROS production in aortas from normotensive and hypertensive rats

**FIGURE 5:**
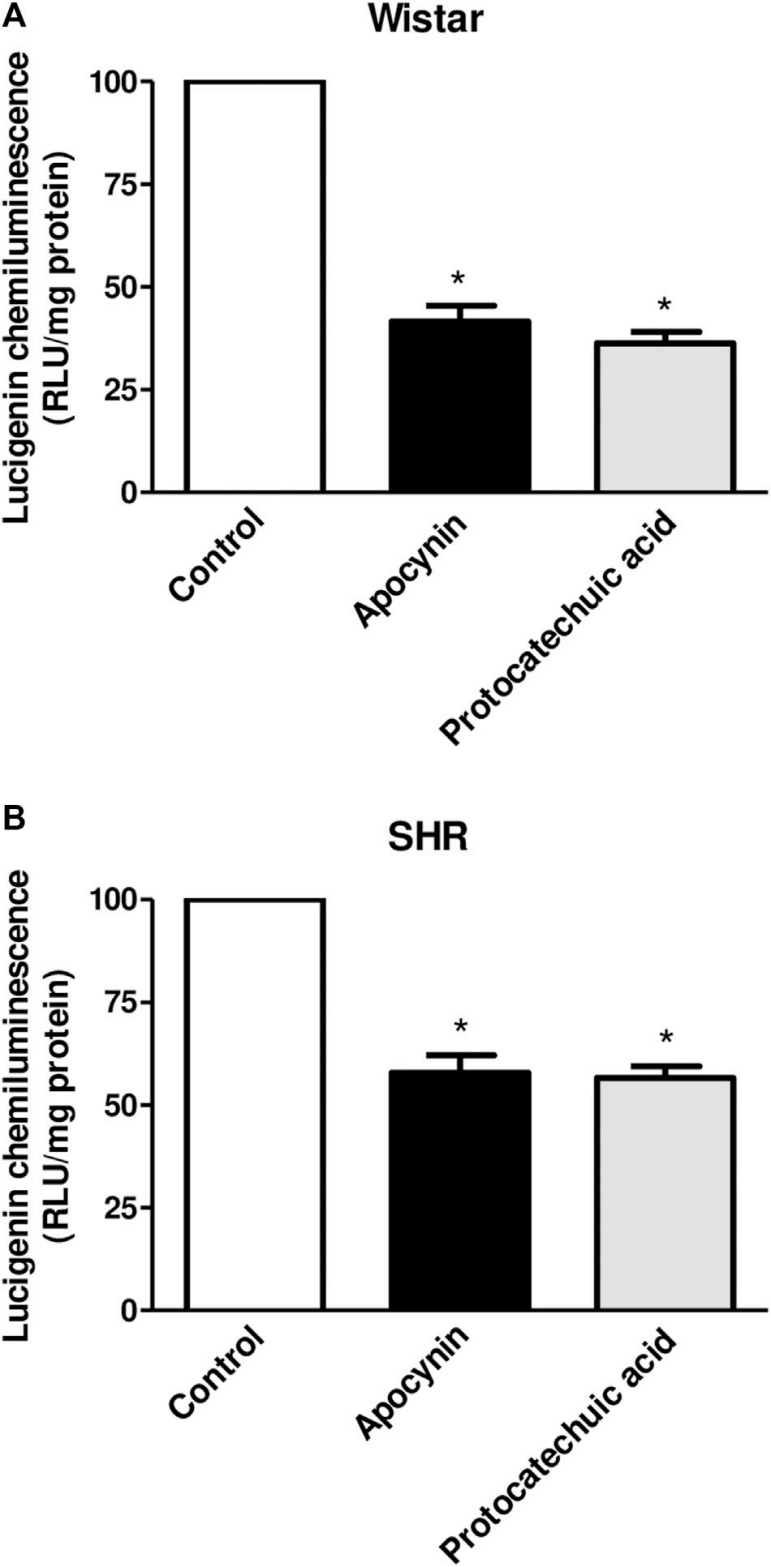
Percentage of lucigenin chemiluminescence, in relative light units (RLU/mg protein), in aortic rings unstimulated (Control, white bars), or stimulated (100 μmol/L) with apocynin (black bars) or protocatechuic acid (grey bars) from Wistar rats **(A)** and SHR **(B)**. Values represent the mean ± SEM of the results, n = 9–10. *p < 0.05 Apocynin and Protocatechuic acid vs. Control.

Lucigenin chemiluminescence was decreased by apocynin and protocatechuic acid in vascular cells of Wistar rats (Figure 5A) and SHR (Figure 5B) compared to their respective control groups, although the reduction in chemiluminescence was less pronounced in hypertensive rats, since this model presents oxidative stress. See description of the results at Supplementary Materials.

### 3.4 Both compounds diminished ROS concentration in HUVEC

**FIGURE 6:**
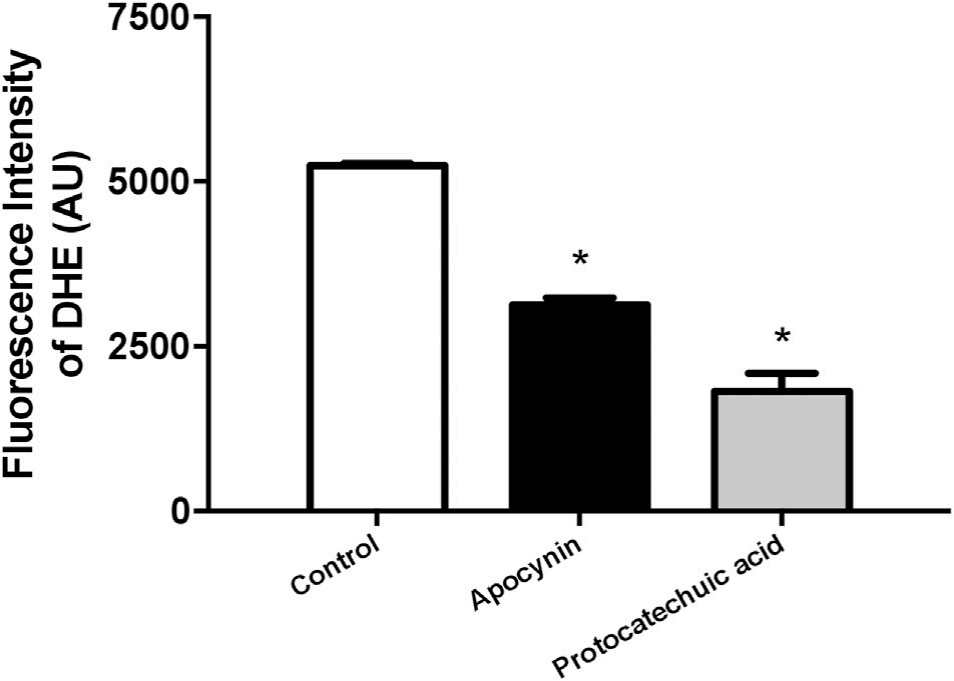
Reactive oxygen species (ROS) concentration, in arbitrary units, in human umbilical vein endothelial cells (HUVEC). Fluorescence intensity was detected by ROS-sensitive probe dihydroethidium (DHE) in cells unstimulated (Control, white bars) or stimulated (100 μmol/L) with apocynin (black bars) or protocatechuic acid (grey bars). Values represent the mean ± SEM of the results, n = 4. *p < 0.05 Apocynin and Protocatechuic acid vs. Control.

In HUVEC, the treatment with apocynin or protocatechuic acid decreased the fluorescence intensity of DHE, a specific probe to detect ROS, when compared to unstimulated cells, indicating that both compounds were able to reduce ROS levels in isolated cells (Figure 6; Supplementary Materials).

### 3.5 Apocynin and protocatechuic acid increased NO^•^ concentration in HUVEC

**FIGURE 7:**
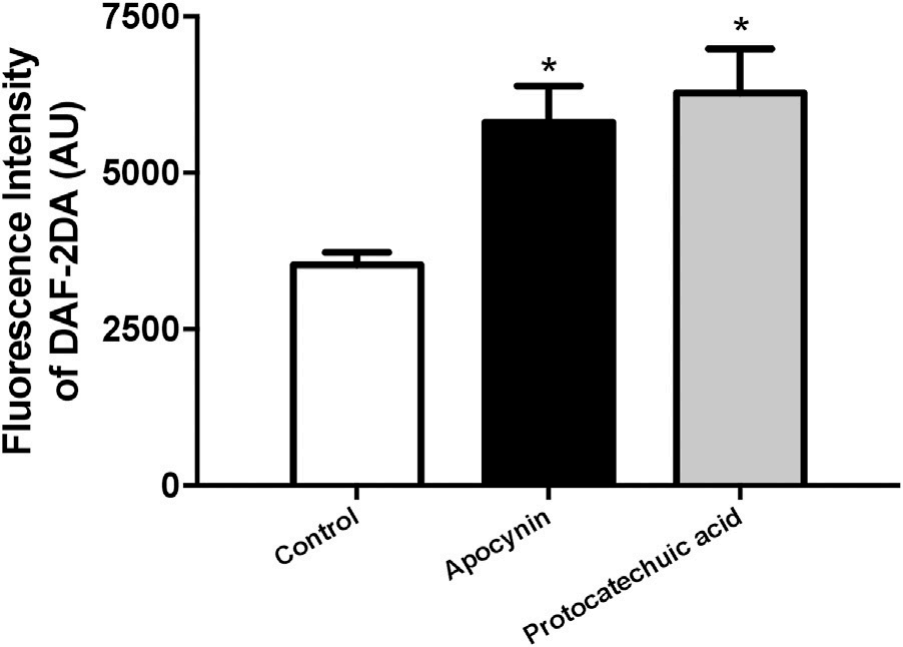
Nitric oxide (NO) concentration, in arbitrary units, in human umbilical vein endothelial cells (HUVEC). Fluorescence intensity was detected by NO-sensitive probe 4,5-diaminofluorescein diacetate (DAF-2DA) in cells unstimulated (Control, white bars) or stimulated (100 μmol/L) with apocynin (black bars) or protocatechuic acid (grey bars). Values represent the mean ± SEM of the results, n = 4. *p < 0.05 Apocynin and Protocatechuic acid vs. Control.

In HUVEC, apocynin and protocatechuic acid increased the fluorescence intensity of DAF-2DA, a specific probe to detect NO, when compared to the control group, showing that both compounds increased the bioavailability of NO in isolated cells (Figure 7; Supplementary Materials).

### 3.6 Apocynin, but not protocatechuic acid, reduced the phenylephrine reactivity in SHR aorta

**FIGURE 8:**
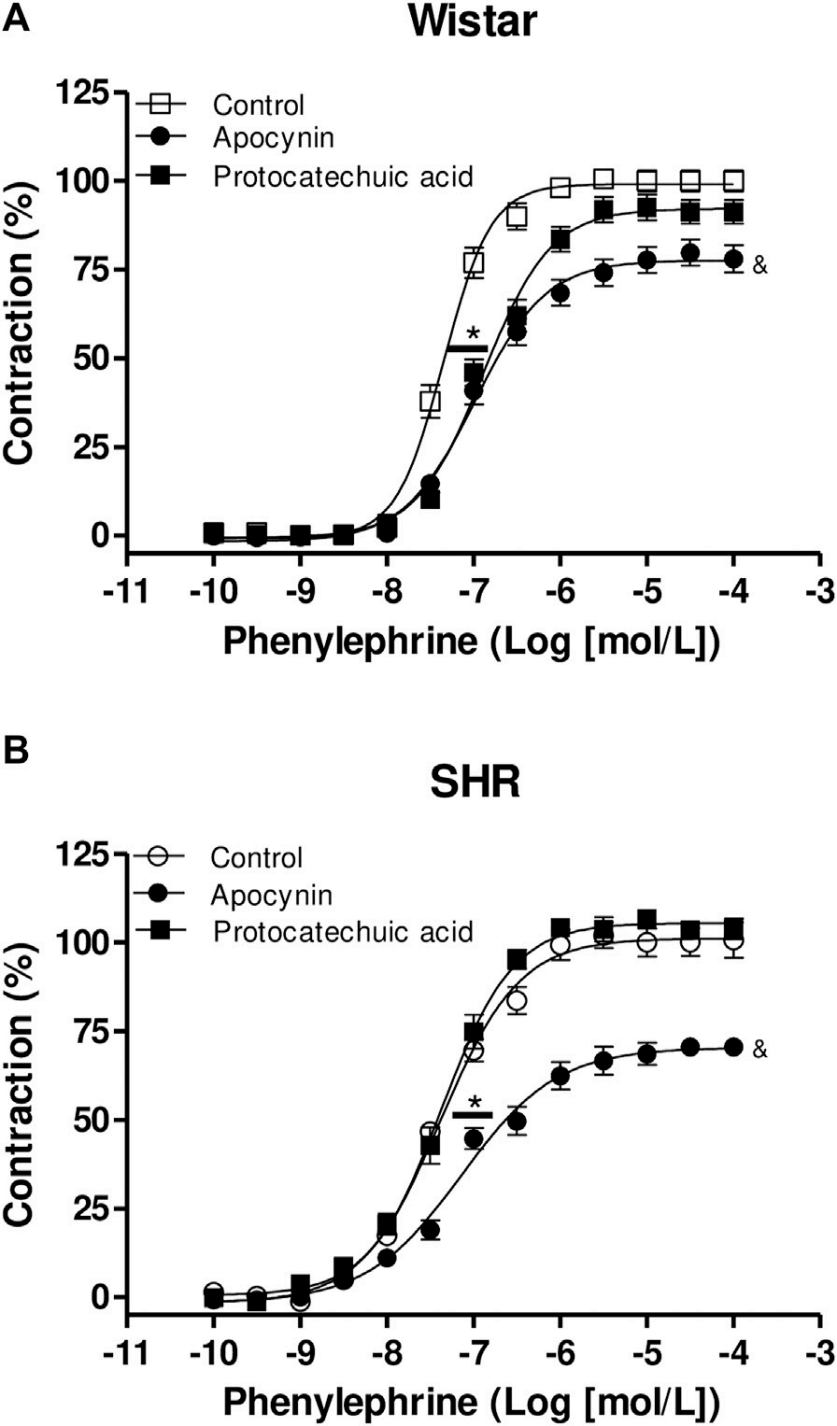
Cumulative concentration-effect curves to phenylephrine (0.1 nmol/L – 100 μmol/L) in aorta rings from Wistar rats **(A)** and SHR **(B)** preincubated (100 μmol/L) with apocynin (black circles) or protocatechuic acid (black squares) or not incubated (Control, white symbols). Values represent the mean ± SEM of the results, in percentage of contraction, n = 4–8. **(A)** *p < 0.05 pD_2_ values of Control vs. Apocynin or Protocatechuic acid, ^&^ *p* < 0.05 Emax values of aortic rings with Apocynin vs. others.

Phenylephrine induced contraction of aorta rings from both rats strains (Figure 8; Supplementary Materials). The incubation of aortas rings with apocynin decreased the maximum effect (Emax) of phenylephrine in aortas from Wistar rats when compared to control curve, but no statistical significance was observed when aortas were incubated with protocatechuic acid. We also observed that the incubation with both compounds decreased the potency (pD_2_) of phenylephrine when compared to the control curve (Figure 8A) in aortic rings of Wistar rats.

Protocatechuic acid did not alter the concentration-effect curves to Phenylephrine in aortic rings of SHR. However, aortic rings of SHR incubated with apocynin showed a lower pD_2_ value to phenylephrine curve when compared to other curves. Moreover, apocynin also decreased the Emax values of phenylephrine curves when compared to the rings not incubated or incubated with protocatechuic acid (Figure 8B).

### 3.7 Apocynin and protocatechuic acid increased the potency of acetylcholine only in aortic rings of SHR

**FIGURE 9:**
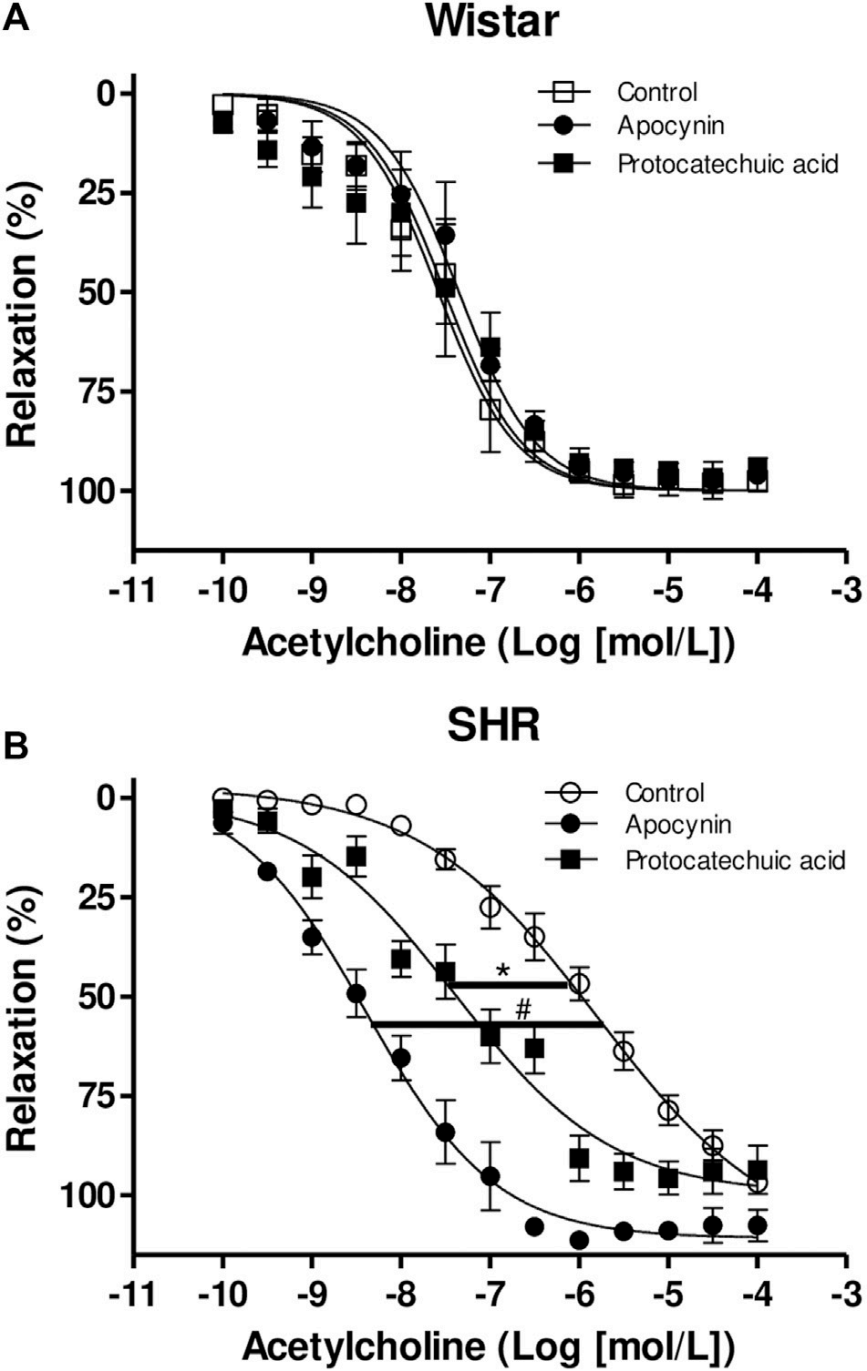
Cumulative concentration-effect curves to acetylcholine (0.1 nmol/L – 100 μmol/L) in aorta rings from Wistar rats **(A)** and SHR **(B)** preincubated (100 μmol/L) with apocynin (black circles) or protocatechuic acid (black squares) or not incubated (Control, white symbols). Values represent the mean ± SEM of the results, in percentage of relaxation, n = 3–7. *p < 0.05 SHR pD_2_ values of Protocatechuic acid vs. Control, ^#^ *p* < 0.001 SHR pD_2_ values of Apocynin vs. Control.

Acetylcholine-induced relaxation is impaired in aortic rings from SHR when compared to aorta rings from Wistar rats (Figure 9; Supplementary Materials). None of the compounds altered the Emax or the pD_2_ of acetylcholine in aorta from Wistar rats (Figure 9A; Supplementary Materials). In aortas from SHR, the compounds did not alter the Emax of acetylcholine. However, we observed that protocatechuic acid and apocynin shifted the acetylcholine-curve to the left, increasing the potency of acetylcholine. When compared, we can observe that apocynin showed greater magnitude effect in SHR aortic rings than protocatechuic acid (Figure 9B).

## 4 Discussion

The main findings of this study are that apocynin had a weak scavenger effect and reduced antioxidant capacity *in vitro* when compared to protocatechuic acid, but in vascular cells, the effects of apocynin are greater than or equal to the effects of protocatechuic acid. Apocynin and protocatechuic acid showed free radical scavenging capacity and an effective inhibitory effect on ROS production. Furthermore, NO^•^ and ROS concentration were modulated by both compounds in isolated endothelial cells, and phenylephrine and acetylcholine vasomotor effects were altered by these compounds.

The reducing ability of a compound may be a significant indicator of its potential antioxidant activity (Kilic et al., 2014). Only higher concentrations (100 μmol/L and 1 mmol/L) of the apocynin and protocatechuic acid compounds exhibited antioxidant capacity. In addition, at the concentration of 1 mmol/L protocatechuic acid had higher antioxidant capacity than apocynin (Figure 2). Antioxidants are defined as molecules with the ability to donate electrons to eliminate the unpaired condition of the radical and neutralize the free radicals. These molecules can directly react with ROS creating less harmful compounds or can regulate the (dys)function of ROS generating enzymes, by inhibiting their activity or expression (Lü et al., 2010). NOX enzymes are the major source of vascular superoxide anion in the organism (Dikalov et al., 2014) and recently we showed that *in vivo* treatment with apocynin decreased the expression of NOX2 in aortas and increased plasma antioxidant capacity in SHR (Perassa et al., 2016; Graton et al., 2019). Protocatechuic acid showed a concentration-dependent antioxidant capacity (Figure 2), as observed previously (Li et al., 2011), and its pharmacological actions were closely correlated with its antioxidant activities (Kakkar and Bais, 2014). Based on these results, we chose the concentration (100 μmol/L) of the both compounds for the subsequent experiments of TBARS, lucigenin chemiluminescence, cell culture and vascular reactivity.

Considering the importance of discriminating between the direct free radical scavenging capacity and the inhibition of NOX activity, the antioxidant potency of the studied compounds was evaluated by three different additional assays. The first was the oxygen radical absorbance capacity (ORAC) assay, which is based on the efficacy of the studied compounds to scavenge peroxyl free radicals (ROO^•^) generated by thermolysis of AAPH at 37°C (Ou et al., 2001). The second method for evaluation of antiradical efficacy was the CAT degradation assay. This method is based on the measurement of the capacity of the studied compounds act as inhibitor of the degradation of the unsaturated fatty acid, eleostearic acid, provoked by ROO^•^ (Laguerre et al., 2008). The third method for evaluation of the antioxidant efficacy was the DPPH method, which is based on the reduction of this stable free radical compound. Figure 3 shows the kinetic profile of fluorescein degradation, triene degradation and antioxidant capacity of the compounds. Each of the compounds were a better scavenger of ROO^•^ compared to Trolox, a water-soluble derivative of vitamin E and protocatechuic acid was more efficient scavenger than apocynin (Table 1). In addition, the inhibitory efficacy of protocatechuic acid was also superior that of apocynin (Table 1). As we can see, when comparing the effects of both compounds by the different methods used, apocynin proved to be a weaker antioxidant than protocatechuic acid.

TBARS is a well-established method for measuring lipid peroxidation, an indicator of oxidative stress (Yagi, 1998) and results are expressed by MDA levels. We did not observe alterations of MDA levels in the Wistar rats aortas incubated with apocynin and protocatechuic acid (Figure 4A). Other studies have also reported no differences following treatment with antioxidants in normotensive rats, presumably due to the absence of oxidative stress in these animals (Tain et al., 2012; Perassa et al., 2016). On the other hand, in aortas from SHR, a model with vascular oxidative stress (Suzuki et al., 1995), apocynin and protocatechuic acid were able to reduce MDA levels (Figure 4B), confirming that these compounds present antioxidant capacity, which can be defined as the ability of a substance to inhibit oxidative degradation, such as lipid peroxidation in vascular cells. The reduction of lipid peroxidation appears to be associated with the antioxidant capacity of these compounds, since other antioxidants, such as ascorbic acid and α-tocopherol, were also able to decrease this marker of oxidative stress (Frei, 1991; Motoyama et al., 1998). It is important to note that although apocynin has a lower antioxidant effect than protocatechuic acid, its effect in reducing MDA levels was similar to that of protocatechuic acid.

Lucigenin is a valid probe for superoxide production detection by enzymatic and cellular sources (Li et al., 1998). Apocynin and protocatechuic acid were able to decrease the lucigenin signal in vascular cells in the aortas from normotensive Wistar rats and SHR (Figure 5), suggesting that both compounds reduces. superoxide production in cells with absence or presence of oxidative stress. From these results, along with the previous ones (Figure 3), we can predict that the compounds here studied showed antioxidants characteristics, due to the ability to scavenging ROS, but also to inhibit their formation.

Among ROS, O_2_^•-^ has a critical importance in the blood vessel due to its rapid reaction with NO^•^ leading to peroxynitrite formation and marked alteration of vascular tone promoting vasoconstriction and altering vasodilatation (Münzel et al., 2002). DHE is an additional probe for the detection of intracellular O_2_^•-^ levels (Chen et al., 2013). To minimize the detection of the nonspecific DHE oxidation product ethidium (E+), we used settings of the excitation (405 nm) and emission (570 nm) to optimize the measurement of the O_2_^•-^ specific product 2-hydroxyethidium (2-OH-E+) (Nazarewicz et al., 2013). Previously it was shown that another phenolic compound known as triazolo pyrimidine VAS3947, the specific NOX inhibitor, was able to inhibit DHE-detectable ROS in SHR aortas (Wind et al., 2010). In addition, apocynin treatment reduced DHE-detectable ROS in fresh aortic endothelial cells of SHR (Perassa et al., 2016). Furthermore, apocynin was able to decrease ROS production in HUVEC due reduced expression and translocation of p47phox (Oliveira et al., 2018). Corroborating these previous studies, we showed that apocynin and protocatechuic acid were able to reduce the fluorescence intensity of DHE in HUVEC.

We next used the membrane-permeable DAF-2 DA probe for detection of intracellular NO^•^ levels. DAF-2 DA is hydrolyzed to DAF-2 and trapped inside the cell after reacting with NO^•^ (Kojima et al., 1998; Zhou and He, 2011). Apocynin and protocatechuic acid increased the fluorescence intensity of DAF-2DA in endothelial cells when compared to control group (Figure 7). Han and others (2019) observed similar results which protocatechuic acid was also able to increase NO^•^ generation in HUVEC. NO^•^ is an important factor that regulates endothelial function, including prevention of platelet adhesion and aggregation, limitation of cholesterol oxidation, vascular smooth muscle cell proliferation and promotes vasodilation (Tousoulis et al., 2012).

In the next step, we analyzed the effects of apocynin and protocatechuic acid on vascular reactivity. Our results demonstrated that apocynin decreased the Emax and potency of phenylephrine in Wistar and SHR aortic rings. In the pathology of hypertension, one of the characteristics is sympathetic hyperactivity (Julius and Nesbitt, 1996). In that sense, an increase in the concentration of circulating noradrenaline and adrenaline was demonstrated in hypertensive animals and patients (Julius and Nesbitt, 1996; Mancia et al., 1999). Phenylephrine is a sympathomimetic drug, therefore, evaluating whether apocynin and protocatechuic acid have the ability to modulate the contraction induced by phenylephrine is an indication that these compounds can be used in pathologies such as hypertension. As observed in our results, apocynin reduced the Emax and potency induced by phenylephrine in aortic rings of normotensive and hypertensive rats. On the other hand, protocatechuic acid reduced the potency of phenylephrine only in the aortic rings of normotensive rats and had no effect on the vessels of hypertensive rats.

It has been reported that phenylephrine-induced contraction activates superoxide anion (Tsai and Jiang, 2010; Silva et al., 2013) and hydrogen peroxide (Silva et al., 2013) production in arteries of rats. It was demonstrated previously by our group that apocynin and its active dimer, diapocynin, promoted hyporeactivity to phenylephrine (Potje et al., 2017), associated with decreased ROS generation in aortic rings of Wistar rats. In addition, the aortic rings from normotensive rats incubated with superoxide scavenger (Tiron) or aortic rings from renal hypertensive rats incubated with peg-catalase, which degrades hydrogen peroxide (H_2_O_2_), showed reduced contractile effect stimulated by phenylephrine (Silva et al., 2013). Moreover, apocynin or VAS2870 (NOX inhibitor) attenuated the contractile effect induced by phenylephrine in rat-tail artery strips. The authors showed that ROS from NADPH oxidase and mitochondria, changed myosin phosphatase-mediated MLC20 phosphorylation through both RhoA/Rho kinase- and CPI-17-dependent pathways, thus regulating α1-adrenoceptor-activated smooth muscle contraction (Tsai and Jiang, 2010). Based on these results, we can conclude that apocynin reduced phenylephrine-induced concentration-dependent contraction in aortic rings of normotensive and hypertensive rats associated with ROS scavenger. However, protocatechuic acid had no anti-contractile effect on the phenylephrine-stimulated concentration-dependent curves even being a more potent ROS scavenger than apocynin as shown by the ORAC assay. More experiments are needed to elucidate the underlying mechanisms of protocatechuic acid on vascular smooth muscle cells.

Endothelial dysfunction plays a central role in the pathogenesis of hypertension (Konukoglu and Uzun, 2017), being characterized by impaired bioavailability/production of endothelium-derived relaxing factors and associated with oxidative stress (Dharmashankar and Widlansky, 2010; Flammer et al., 2012). In addition, endothelial dysfunction favors the thrombotic and inflammatory process in blood vessels. SHR aorta have impaired acetylcholine-induced relaxation due to endothelial dysfunction (Potje et al., 2019). As observed in this present study, acetylcholine promoted relaxation in aortic rings from Wistar rats and SHR, but we can clearly see the impairment of potency induced by acetylcholine in aortic rings of SHR, confirming endothelial dysfunction. Subsequently, we observed that apocynin and protocatechuic acid increased the potency of acetylcholine only in aortic rings of hypertensive rats, in other words, these compounds were efficient in improving endothelial function. In aortic rings from normotensive rats, which do not show endothelial dysfunction, both compounds did not alter acetylcholine-induced relaxation.

It was reported that the chronic treatment with apocynin prevented hypertension in fructose fed rat models (Unger and Patil, 2009). Later, our group also reported that the chronic treatment with apocynin prevented development of hypertension in SHR (Perassa et al., 2016). Moreover, in the aortic rings of fructose-fed rats, apocynin increased the Emax of the acetylcholine curves, but the Emax values were not similar to the control curve, indicating that apocynin improved endothelial function, but did not recover the basal endothelial function (Unger and Patil, 2009). However, in our previously study, the chronic treatment with apocynin shifted the acetylcholine curve to the left in aortas of SHR and these curves were similar to the acetylcholine-curves constructed in the aorta of normotensive rats, confirming that apocynin recovered endothelial function in vessels of SHR (Perassa et al., 2016). Therefore, it is clearly shown that apocynin has a physiological effect on the smooth muscle vascular cell.

Different groups have shown different effects of protocatechuic acid. The chronic treatment (20 weeks) with protocatechuic acid in the apolipoprotein E (ApoE)-deficient mouse model was able to decrease the aortic vascular cell adhesion protein 1 (VCAM-1) and intercellular adhesion molecule 1 (ICAM-1) expression and plasma-soluble VCAM-1 and ICAM-1 levels, inhibiting atherosclerosis development (Wang et al., 2010). In addition, the HUVECs treated with protocatechuic acid (100 μmol/L) showed a reduction of two biomarkers of oxidative stress, 3-nitrotyrosine (3-NT) and 8-hydroxydeoxyguanosine (8-OHdG), and the nitric oxide levels were increased (Han et al., 2019). In our present study, we show that protocatechuic acid has a physiological effect, increasing the relaxation induced by acetylcholine in aortic rings of SHR, but the pathway mechanism of action needs to be investigated.

Therefore, O_2_^•-^ scavenging appears to be useful in the restoring of endothelium-dependent dilatation as demonstrated in SHR blood vessels (Figure 9B). In addition, even though SHR showed increased ROS production, both compounds were able to oxidize the ROS and decrease the lucigenin chemiluminescence. Our findings are in accordance with previous results showing the beneficial effects of antioxidants on the biological activity of NO^•^ and endothelium-dependent relaxation (Carr and Frei, 2000). As endothelial dysfunction is an independent predictor of cardiovascular risk (Schiffrin, 2008), these data provide new information on the benefits of using drugs that act as antioxidants or inhibit the production of ROS, particularly in disorders with marked endothelial dysfunction. Taken together, the compounds tested in this study should be considered in the treatment of these vascular diseases, mainly apocynin, which seems to present a higher efficacious therapeutic in cases of endothelial dysfunction as observed in this study and reported by other groups.

## 5 Conclusion

In *vitro* assays, protocatechuic acid showed greater ability to act as a ROS scavenger and to have a greater antioxidant capacity. However, when we tested the compounds in isolated blood vessels, apocynin showed a more effective pharmacological function, since it reduced the contraction induced by phenylephrine with greater magnitude in aortas of normotensive and hypertensive vessels and it also improved the endothelium-dependent relaxation in SHR, a model of endothelial dysfunction.

Our study reinforces the importance of using substances that combine the ability to scavenge and/or prevent the ROS generation, and the reestablishment of NO^•^ bioactivity. Based on the data presented here apocynin and protocatechuic acid are candidates for future therapeutic approaches of cardiovascular pathological conditions where oxidative stress is a key feature. Future studies involving the *in vivo* treatment should be performed to evaluate other potential effects of these drugs, as well, for the better description and elucidation of mechanism of action of these drugs, in order to check potential improvements on the cardiovascular function, mainly in hypertension.

## Data Availability

The original contributions presented in the study are available at https://repositorio.unesp.br/handle/11449/237174 and included in the article/Supplementary Materials, further inquiries can be directed to the corresponding author.
