# Supplementary material for: Comparative study between apocynin and protocatechuic acid regarding antioxidant capacity and vascular effects
Source: Front Physiol. 2022 Nov 15;13:1047916. doi: 10.3389/fphys.2022.1047916 (PMC9707364; doi:10.3389/fphys.2022.1047916)
Supplement: Supplementary file 1 [file DataSheet1.PDF]

Data List

Antioxidant capacity of the studied compounds

Lower concentrations of the apocynin and protocatechuic acid did not demonstrate significant antioxidant capacity (0.1 nmol/L to 10 µmol/L). Antioxidant capacity was observed at concentrations more than 100 µmol/L (Apocynin: 0.24 ± 0.001; Protocatechuic acid: 0.39 ± 0.005; mmol/L/ $FeSO_4$). In addition, at the concentration of 1 mmol/L protocatechuic acid (3.85 ± 0.002) had higher antioxidant capacity than apocynin (1.17 ± 0.05) (Fig. 2).

3.2. Evaluation of direct free radical scavenging capacity and the inhibitory effect on NOX

Using the oxygen radical absorbance capacity (ORAC) assay, protocatechuic acid showed at least 1.5-fold greater ability to act as a scavenger than apocynin (Fig. 3A and B). Through the conjugated autoxidizable triene (CAT) degradation assay, protocatechuic acid showed an inhibitory effect six times greater than that of apocynin (Fig. 3A and C). Furthermore, by the DPPH method, protocatechuic acid was significantly more effective than apocynin as it needed at least a 700-fold lower concentration to scavenger DPPH compared to apocynin (Fig. 3A).

3.3. Apocynin and protocatechuic acid can decrease oxidative stress biomarkers in SHR

Apocynin (53.59 ± 3.95, n=7, nmol MDA/mg of protein) and protocatechuic acid (48.17 ± 3.59, n=6, nmol MDA/mg of protein) did not change MDA levels in aortic rings from Wistar rats, values remaining similar to unstimulated aortic rings (Control: 52.42 ± 3.90, n=6, nmol MDA/mg of protein) (Fig. 4A). On the other hand, apocynin (32.24 ± 1.71, n=5, nmol MDA/mg of protein) and protocatechuic acid (28.48 ± 3.94, n=5, nmol MDA/mg of protein) were able to reduce MDA levels in aortic rings from SHR when compared to unstimulated rings (Control: 65.36 ± 3.92, n=5, nmol MDA/mg of protein) (Fig. 4B).

3.4. Both compounds can decrease ROS production in aortas

Lucigenin chemiluminescence was decreased by apocynin and protocatechuic acid in vascular cells of Wistar rats (Apocynin: 41.5 ± 3.9; Protocatechuic acid: 36.3 ± 2.7; RLU/mg of protein, n = 9-10, Fig. 5A) and SHR (Apocynin: 57.9 ± 4.2; Protocatechuic acid: 56.6 ± 2.8; RLU/mg of protein, n = 9-10, Fig. 5B) compared to their respective control groups.

3.5. Both compounds diminished ROS concentration in HUVEC

In HUVEC, apocynin (3131 ± 103.2 AU, n = 4) and protocatechuic acid (1813 ± 278.5 UA, n=4) decreased the fluorescence intensity of DHE, when compared to unstimulated cells (control: 5238 ± 32.6 AU, n = 4) (Fig. 6).

3.6. Apocynin and protocatechuic acid augmented NO• concentration in HUVEC

In HUVEC, apocynin (5808 ± 548.1 UA, n = 4) and protocatechuic acid (6281 ± 701.3 UA, n = 4) increased the fluorescence intensity of DAF-2DA when compared to control group (3530 ± 198.1 UA, n = 4) (Fig. 7).

3.7. Effect of the compounds on the phenylephrine-induced contraction

Phenylephrine induced contraction of aorta rings from both rats strains (Fig. 8). The incubation of aortas rings with apocynin decreased the maximum effect (Emax) of phenylephrine in aortas from Wistar rats when compared to control curve (Control: 100.00 ± 2.99; Apocynin: 78.02± 3.77, %, n = 5-6), but no statistical significance was observed when aortas were incubated with protocatechuic acid (91.25 ± 3.37, %, n = 6). We also observed that the incubation with both compounds decreased the potency (pD_2) of phenylephrine when compared to the control curve (Control: 7.3 ± 0.1; Apocynin: 6.9 ± 0.12; Protocatechuic acid: 6.9 ± 0.10) (Fig. 8A) in aortic rings of Wistar rats.

Aortic rings of SHR incubated with apocynin showed a lower pD_2 value to phenylephrine curve when compared to other curves (Control: 7.38 ± 0.1; Apocynin: 7.13 ± 0.1; Protocatechuic acid: 7.36 ± 0.2; n = 4-8). Moreover, apocynin also decreased the Emax values induced by phenylephrine when compared to the rings not incubated or incubated with protocatechuic acid (Control: 100.67 ± 5.00; Apocynin: 70.56 ± 0.90; Protocatechuic acid: 100.80 ± 2.95; %, n = 4-8) (Fig. 8B).

3.8. Acetylcholine-induced relaxation in aortas incubated with the compounds

Acetylcholine-induced relaxation is impaired in aortic rings from SHR (6.09 ± 0.13, n = 7) compared to curves constructed in aorta rings from Wistar rats (7.5 ± 0.21, n = 3-4) (Fig. 9). None of the compounds altered the Emax (Control: 97.5 ± 1.25; Apocynin: 96.0 ± 4.12; Protocatechuic acid: 94.2 ± 1.26; % of relaxation, n = 3-4) or the pD_2 (Control: 7.5 ± 0.21; Apocynin: 7.3 ± 0.15; Protocatechuic acid: 7.5 ± 0.31; n = 3-4) of acetylcholine in aorta from Wistar rats (Fig. 9A). In aortas from SHR, the Emax of acetylcholine was not altered by the compounds (Control: 96.8 ± 1.56; Apocynin: 107.6 ± 3.94; Protocatechuic acid: 93.6 ± 6.12; % of relaxation, n = 3-7). However, we observed that protocatechuic acid (7.2 ± 0.38; n = 4-3) and apocynin (8.1 ± 0.39, n = 3) shifted the acetylcholine-curve to the left, this means that both compounds increased the potency of acetylcholine compared to control curve (6.09 ± 0.13, n = 7), and this displacement showed greater magnitude in aortic rings incubated with apocynin (Fig. 9B).
